# MICROBIOME ROLE IN COGNITIVE IMPAIRMENT: MIAGB COHORT INSIGHTS

**DOI:** 10.1093/geroni/igae098.2629

**Published:** 2024-12-31

**Authors:** Sidharth Mishra, Lauren Buddendorff, Julia Hoover, Rohit Shukla, Vivek Kumar, Shalini Jain, Hariom Yadav

**Affiliations:** University of South Florida, Tampa, Florida, United States; University of South Florida, Tampa, Florida, United States; University of South Florida, Tampa, Florida, United States; University of South Florida, Tampa, Florida, United States; University of South Florida, Tampa, Florida, United States; University of South Florida, Tampa, FL, USA, Florida, United States; University of South Florida, Tampa, Florida, United States

## Abstract

Aging population is increasing, leading to a rise in aging-related conditions, specifically diseases like Alzheimer’s disease and its related dementia (ADRD), which have poor prognosis and lack prevention and treatment strategies. Although we do not fully understand the complex pathology of ADRD, emerging evidence indicates that abnormalities in the gut microbiome contribute to ADRD pathology, but how this occurs remains largely unknown. Utilizing samples and datasets from a multi-site and comprehensive clinical study called the Microbiome in aging Gut and Brain (MiaGB) consortium, of comprising 30 older adults with mild cognitive impairment (MCI) and 43 age- and sex-matched cognitively healthy controls, we found that older adults with MCI have higher levels of circulating gut permeability markers (lipopolysaccharide [LPS] binding protein [LBP] and sCD14) and systemic inflammation markers (IL-6, CRP, and IL-1β). Increased gut permeability (leaky gut) is an understudied source of systemic inflammation as it allows pro-inflammatory ingredients including LPS and microbes from the gut lumen to enter the bloodstream, instigating systemic inflammation. Interestingly, increased LPS and TLR4 activation were found in the circulation of MCI participants compared to controls. Furthermore, we discovered that gut microbial metabolite- ethanolamine utilization pathways were decreased in MCI compared to controls. These abnormalities are significantly associated with reduced cognitive function (MoCA scores) in older adults. These findings provide mechanistic insight into how abnormalities in the gut microbiota, with reduced ethanolamine utilization, result in ethanolamine accumulation in the gut, inducing leaky gut, which in turn activates systemic inflammation, ultimately accelerating cognitive decline in older adults.

